# Reliability and reproducibility of trans-valvular flow measurement by 4D flow magnetic resonance imaging in acute myocardial infarct patients: two centre study

**DOI:** 10.1186/1532-429X-18-S1-P36

**Published:** 2016-01-27

**Authors:** Pankaj Garg, Mariella E Hassell, David P Ripley, Laura E Dobson, Peter Swoboda, Tarique A Musa, Bara Erhayiem, Philip Haaf, John P Greenwood, Robin Nijveldt, Jos J Westenberg, Rob J van der Geest, Sven Plein

**Affiliations:** 1 Multidisciplinary Cardiovascular Research Centre & The Division of Cardiovascular and Diabetes Research, Leeds Institute of Genetics, Health & Therapeutics, University of Leeds, Leeds, UK; 2 Department of Cardiology, Academic Medical Center, University of Amsterdam, Amsterdam, Netherlands; 3 Department of Cardiology, VU University Medical Center Amsterdam, Amsterdam, Netherlands; 4 Division of Image Processing, Leiden University Medical Center, Leiden, Netherlands

## Background

Time resolved 3-dimensional 3-directional phase contrast (PC) Magnetic Resonance Imaging (4D flow MRI) has advantage over 2-dimensional PC-MRI as it allows accurate quantification of stroke volumes (SV) using retrospective valve tracking and subsequent reconstruction of the transvalvular flow velocity throughout the cardiac cycle. However, the reliability of this method has not been tested between centres with different observers or in acute myocardial (AMI) patients with regional wall motion abnormality. We hypothesize that the SV assessed by 4D flow MRI using valve tracking flow assessment through the mitral valve (MV) and the aortic valve (AV) is reproducible among two observers from different sites.

## Methods

Fifteen AMI patients underwent CMR at 1.5T (Ingenia CV, Philips Healthcare, Best, The Netherlands). CMR Protocol included: 2-chamber, 3-chamber, 4-chamber cines and 4D flow MRI with isotropic voxel size (3 × 3 × 3 mm3), parallel imaging (SENSE 2), velocity sensitivity Venc 150 cm/s in all three directions and using echo-planar imaging (EPI) to factor of 5 for read-out acceleration. Free breathing was allowed and no respiratory motion correction was used. Retrospective gating was used and 30 cardiac phases were reconstructed. Images were analysed by two assessors (MH and PG) from two sites blinded to each other. Retrospective valve tracking with measurement planes positioned perpendicular to the inflow direction on 2-, 3- and 4-chamber cines was used to calculate SV. Background correction was used from velocity sampled in the myocardium.

## Results

Mean MV SV was 68 ± 15 ml (PG) and 67 ± 17 ml (MH) (p = 0.90). Mean AV SV was 70 ± 20 ml (PG) and 73 ± 14 ml (MH) (p = 0.96). Eight (53%) patients had mitral regurgitation, four (27%) had aortic regurgitation. All regurgitation was graded as trivial/mild (MR fraction - 6.7 ± 2%; AR fraction - 1.9 ± 4%). A Bland-Altman analysis for both observers for the SV assessment using the MV and the AV were as follows: PG: mean bias of 2.5 ml, 95% CI -7 to 12, MH: mean bias 5.5 ml, 95% CI -7.8 to 18.9. Coefficient of variation (CV) of inter-observer variability for MV SV was 6.3% and the concordance correlation coefficient was 0.93 CI (0.81-0.97) with accuracy of 0.99. CV of inter-observer variability for AV SV was 6.4% and the concordance correlation coefficient was 0.90 CI (0.72-0.96) with accuracy of 0.98.

## Conclusions

4D flow MRI with retrospective valve tracking provides reliable assessment of transvalvular flow in AMI patients with high inter-observer agreement between sites.

**Figure 1:**
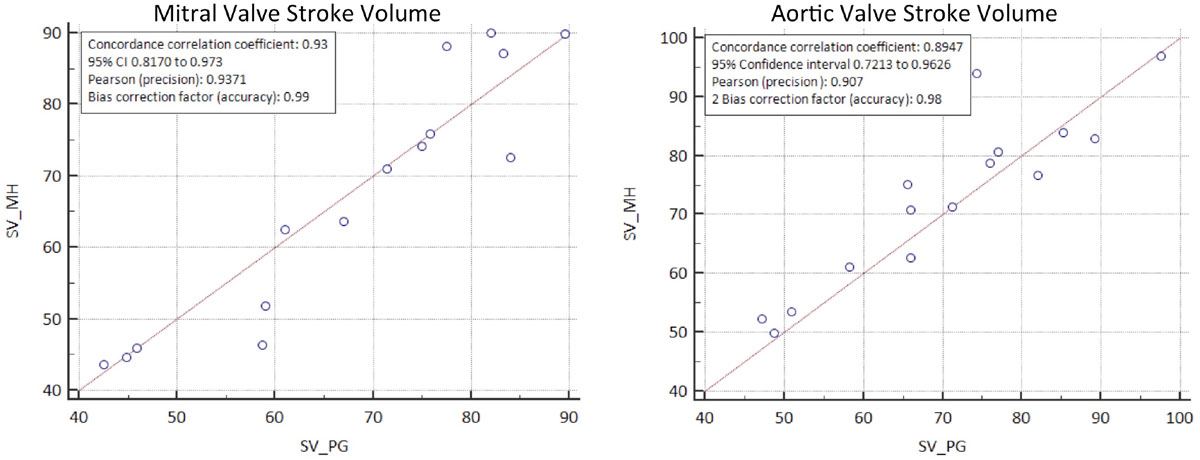
**Scatter diagram for MV and AV stroke volume demonstrating inter-observer reproducibility**.

